# The bioethics of skeletal anatomy collections from India

**DOI:** 10.1038/s41467-024-45738-6

**Published:** 2024-02-24

**Authors:** Sabrina C. Agarwal

**Affiliations:** Department of Anthropology, University of California, Berkeley, Berkeley, CA 94704 USA

**Keywords:** Skeleton, Ethics, Biological anthropology

## Abstract

Millions of skeletal remains from South Asia were exported in red markets (the underground economy of human tissues/organs) to educational institutions globally for over a century. It is time to recognize the personhood of the people who were systematically made into anatomical objects and acknowledge the scientific racism in creating and continuing to use them.

## Main

Human remains historically sourced from South Asia (India) are the most ubiquitous contemporary anatomical collections used globally^1,2^. Many were likely obtained illegally in the red market, i.e., the lucrative and underground economy of trade in human tissues such as organs, bones, and hair. However, almost no attention has been paid to the ethics of working with these human remains. Human skeletons from India were the primary global source of human bone for over 180 years, with the exportation of skeletons that began under British colonial rule and expanded to global exportation after Indian independence^1,3^. In response to increasing concerns by human rights groups over the unethical practices of how bones were being obtained, in 1985 the Supreme Court of India banned the export of human bones (and other tissues) under the National Import/Export Control Act^4^. However, many international and national groups lobbied to end the ban, and illegal exports continued, and likely still continue, to some extent^4–6^. Prior to the ban in 1985, it is estimated that up to 60,000 skeletons/year were exported out of the country^1^. These historical skeletal human remains continue to train generations of biomedical practitioners and biological/forensic anthropologists globally, with hundreds of thousands of red market skeletons in the classrooms of Western countries today. These global collections of skeletal bodies from India are relevant not only to conversations of bioethics, but also those of colonialism and scientific racism. However, we cannot consider how they should be ethically treated without understanding the historical context of how these skeletal bodies came into being in the first place, and acknowledging why they continue to be used in labs and classrooms around the world. I detail here the historical context of the anatomical bone trade in India, the ethical concerns, and the potential solutions from my own positionality as a South Asian bioanthropologist.

### Why India? Biopower, necropolitics, and the colonial rule

The story of how India came to be the largest producer and exporter of prepared anatomical human skeletal material, begins during British colonial rule and is set in the history of medicine and anatomy itself. During the growth of medical education in 18th and 19th century Europe and North America, there began to be a greater need for bodies to be used for anatomical training and dissection^1,7^, and thus the widespread practice of grave robbing of the impoverished and racially disenfranchised^7,8^. Despite public outrage that led to anatomy riots, it was not until widespread anger after the infamous case of Burke and Hare, who murdered individuals and sold their bodies to anatomist Robert Knox, that the 1832 Anatomy Act was passed in Great Britain. The 1832 Act reduced grave robbing and included the first provisions for willed bodies, but primarily led to the increased and legal use of “unclaimed bodies”—bodies of prisoners, those historically deemed poor and “destitute”, and/or those that were historically considered to be “psychiatric” patients^7,8^. However, the demand and shortfall for bodies in dissection rooms continued, and as such, Britain looked to its colonies, particularly as the British began medical education in India.

In India, the British utilized and adapted the caste system for the procurement and preparation of bodies. In the dissection rooms of hospitals and colleges, the British relied upon the community members called Doms^3,9^. Doms, were/are widespread in many areas of India, particularly in Bengal and Bihar, and represent one of the lowest of all castes in India (despised even by Dalits, those considered outside the traditional caste hierarchy that were historically called “Untouchables”). They fulfill tasks that are considered particularly polluting and defiling, like removing animal carcasses and carrying and tending to the human dead in burning or cremation grounds called ghats^3^.

Both Doms and Aghoris, one of the most extreme and controversial sect of Hindu holy men who often dwell near cremation grounds, are popularly known as exoticized Eastern practitioners of death rituals (tourists often flock to the city of Varanasi, formerly known as Benares, to bear witness to their death rituals)^10^. As such, it is perhaps tempting to consider Indian, specifically elaborate Hindu rituals of death and the everyday handling of the dead by certain castes as naturally extending to the business of preparation and exportation of anatomical human remains. However, Doms were pressed into the service of human dissections by British rulers, and a deep loathing and suspicion of cutting up bodies in India persisted during the 19th century, even amongst Doms themselves^3^. The establishment of Western medicine in India, and with it the practice of the collection and dissection of human remains, was integral to the colonizing process. While medical establishments were first set up in India to serve military personnel and British civilians, by the mid-19th century, medical services were initiated to provide care for the Indian populations, too, and to improve public health, with the eventual establishment of the first medical school (Calcutta Medical College) in 1835 that started Western medical education in India^11^. Western medicine established the dominance of scientific thought and practice that eclipsed and/or marginalized Indigenous systems of being and health^12,13^. The introduction of anatomical dissection became a key component to colonial hegemony, by establishing not only the education of anatomy and Western perceptions of the body^13^, but also through using Indian bodies (both the dead and the use of Doms to procure bodies) as a site of colonizing power^3,13^. The scale is appreciated by the account that in the 8 years between 1837 and 1844, some 3500 bodies were dissected at the Calcutta Medical College^14^. The corporality of colonialism in India stood in the space of medicine as a place to use the body for discipline, control, authority, and legitimacy^3^.

Moreover, in the colony of India there was unparalleled opportunity for collecting human remains. By the 1850’s the Calcutta Medical College alone was processing nine hundred skeletons a year for shipment abroad^1^. This number would rapidly multiply in the following decades, most likely fueled during the various periods of pandemics and famine. The export of anatomical specimens from Kolkata particularly expanded during World War Two and following the 1943 Bengal Famine^15^. The famine in the Bengal province of British India (now Bangladesh, West Bengal, and eastern India) claimed the lives of up to 3 million people to starvation and disease, with a disruption in the local economy and destruction of agrarian communities and families^16^. The Bengal Famine is regarded as the only one in modern Indian history that was not simply a result of serious drought; it has instead been largely blamed on Churchill-era British denial policies that utilized a “scorched earth” response related to the Japanese occupation of Burma^17^. World War II geopolitical calculations and racist policies by British authorities were responsible for the rice shortage famine, and the disregard for the hunger and deaths of millions^18^.

Foucault has termed the social and political power used to achieve the control of people’s bodies and lives, as biopower^19^. An extension of biopower is necropolitics, as developed by theorist Achille Mbebme^20^, that expands how socio-political power can be used to dictate not just how others live but also how others die or live suspended in precarious conditions. Both concepts aid in understanding how the practice of medicine, anatomy, and the slow death from starvation in colonial India intertwined with the exertion of authority and control on Indian bodies. The capitalization of famine bodies is chronicled in a 1943 Life Magazine article^21^ on a bone trader from Kolkata who exported skeletons from victims of famine and the American anatomical preparators who received such exports. Similar traders were well known in Kolkata for supplying skeletal material anatomical preparation houses in the UK throughout the 1930s and the decades following the famines^15,21^. These practices can be interpreted as representing the continuity of colonizing biopower on the Indian body, which was wielded not only over the living and the dead, but also over victims of famine that represented the moribund, whose prolonged dying and suffering was easily disregarded and would eventually yield further skeletonized bodies.

### Postcolonial power: supply and demand

The export of bodies continued in complex ways after British rule. India continued the bone trade they had begun under colonial power, first with continued accommodation and participation, and eventually appropriating the practice entirely by serving as middlemen to wealthier countries still seeking inexpensive anatomical exports from India. Following the independence of India, the export of human skeletons continued to grow exponentially with the demands from medical schools and students abroad. It is estimated that Kolkata exporters traded almost 1.5 million dollars’ worth of skeletons just prior to the ban in 1985, with other estimates as high as 5 or 6 million^2^. The Chicago Tribune estimated that 60,000 skulls alone were shipped from Kolkata^1^. A conservative estimate of 40 years of exports of similar numbers from Indian Independence in 1947 to 1985 arrives at an estimate of 2.4 million Indian skeletons and skulls. This does not even account for bone specimens collected for pathological or phrenological studies in the hundred years prior to Independence, which are well documented in museum collections globally, or the use of skeletons within India itself^22,23^.

India continues to be a leading producer of anatomical skeletons for use primarily within India, and a global exporter of human bodies for medical education^24^. The 1949 Anatomy Act provides for the supply of unclaimed bodies of deceased persons to hospitals and medical teaching institutions for the purpose of anatomical examination, dissection, and removal of transplant organs. Bodies are deemed “unclaimed” anywhere between only 24–72 h after death depending on the state^25^. At the time of independence in 1947, there were 23 medical colleges with an annual admission of 1000 students^9^. With the rapid increase in privately-owned medical schools, India now has the most medical colleges in the world, with a total of 63,250 students enrolled for medical education in the academic year 2018–19^26^ and 606 medical/specialty schools as of 2020^27^. Skeletons and soft tissue bodies are used for learning basic topographical anatomy and modern surgical practice^9^ and to keep up the tradition of each entering medical student purchasing their own study skeleton as a status symbol along with a stethoscope^22^, which continues to fuel the industry in India.

### The transformation and the materiality of the study skeleton

Medical ethics typically deals with research on living subjects, with informed consent, particularly the recognition of human dignity and individual autonomy, the cornerstone of ethical practice^28,29^. However, the concept of informed consent is not easily transferable to anatomical bodies. This is partly due to the lack of transferability of the Western concepts of dignity and autonomy, but also to the fact that the dead cannot speak for themselves and, in some ways, occupy an ambiguous status as both object and subject^29^. The contemporary ethical use and curation of Indian anatomical remains is particularly difficult to envision through the lens of two centuries of fluid power dynamics that grew from colonial practices and continues with the production of anatomical skeletons domestically in India today. I argue that part of our difficulty also lies in the materiality of the crafted skeletal bodies themselves. While individuals that were taken and skeletonized over the course of 200 years came from varying villages, religions, ghats, and cemeteries, they were transformed into uniform anatomical objects. The success of the bone trade in India was built on its renowned ability to produce standardized specimens. Bodies were processed meticulously: corpses were often wrapped and anchored in rivers to be dismembered naturally by bacteria and fish; crews of hands would scrub and boil the bones in water and caustic soda to dissolve remaining flesh; and then sun and hydrochloric acid soaks would be used to produce medical-grade gleaming white skeletons with high quality distinguishable anatomical landmarks^1^. While early exports to Britain and colonies such as Australia appear to have been disarticulated haphazardly^23^, later exports to North America were strikingly similar and unmatched by exporters from China or Eastern Europe. These export-quality teaching skeletons are notably uniform. While both males and females are usually represented, most are similar in age, they have intact teeth, and little or no pathology or trauma. The selective and transformative process was explicitly crafted to rid signs of the individual, and specifically the *brown* individual. Unlike early anatomical phrenological specimens that were assigned or retained partial histories because they were valuable to comparative studies of race and human variation, the value of these Indian bodies came from erasing any history, in making them geographically displaced. Biomedical students utilize these bodies as nothing more than objects of the inert skeleton, using them to recognize and memorize parts and reconstruct topographic anatomy. Unlike soft tissue bodies, they are rarely linked to physical markers of their life as people, such as sex or age, and there is no effort to think about where they are from, unlike recently acquired anatomical bodies obtained through consented donor programs. While biological anthropologists do make use of these skeletons to estimate demographics, they are typically used in the classroom to gain the basic skills of anatomical identification that, ironically, are applied to better understand the life experiences of past people excavated from more valued archeological settings. In homogenizing these bodies as simply South Asian “teaching skeletons”, we are all guilty of collapsing not just two centuries of diverse histories and biographies^30^ but also willfully losing all aspects of their social identity and humanity.

Some contemporary scientists have suggested that the export of skeletons from India was a “convenient” arrangement, presuming that the body was not valued after death due to Hindu beliefs in the reincarnation of the soul^23^. This demonstrates the ignorance that persists today of Hindu religious beliefs and funerary customs. Hindus place great significance on the release of the soul only through the breaking of the skull on the final embers of the cremation pyre^31^. Further, Indians in the 19th and 20th centuries did not voluntarily choose to be made into an anatomical study skeleton instead of burial or cremation without some duress. While there is little documentation on the acquisition of the bodies used to create skeletal exports, there are detailed accounts of the business just prior and following the ban. There are accounts of intact bodies being taken from the Ganges River from impoverished families that were not able to conduct cremation. Further, there are several accounts in Bengal of the widespread robbing of skeletons from cemeteries before and after the ban, and/or the purchase or agreement to take bodies prior to death from families that had no resources for burial or cremation^1,23^. Here, it is also important to note that the heart of the bone trading industry was in West Bengal– which has a large Muslim community, not just Hindu^1^.

### What should we do now with the anatomical skeletal remains from India?

In the past decade, researchers have demonstrated how ethical engagement with anatomical collections and their history can continue to inform the political present^32,33^, and they have recently called for the creation of ethical guidelines and policies to deal specifically with museum collections^34,35^. Many questions have also been raised on how best to treat and deal with specifically the South Asian skeletal remains that are found in historical anatomical collections around the globe^23,36–38^. However, the bioethical discussion surrounding the curation of skeletal collections from India urgently needs to take into consideration the unique history of commodification that spans colonial rule, decades of bone traders in Independent India, and the contemporary body brokers of the unclaimed. How do we acknowledge the structures of violence that undoubtedly encircled these people in life and death as objects of textbook anatomical landmarks, and is there a way to return what was taken from these South Asian ancestors?

The Indian skeletons that make up our global collections were never willed or donated, at least in the sense of what we deem altruistic biomedical donation, or with contemporary standards of valid consent. Their continued use is the result of our historic complicity and scientific opportunism. The bodies of Indians were taken, their flesh literally stripped and dissolved to remove and reconstitute their identity as nothing more than an anatomical teaching skeleton, a task done so well that their humanity remains largely forgotten or discounted. In 2022 *The Anatomical Record*, the official publication of the American Association for Anatomy, published a special issue on the changing face of the field with a particular focus on the foundation of colonial science, racism and ethics in anatomy^39^. Not a single paper in the issue mentions the millions of Indian bodies that have been formative in skeletal anatomy education. Only now as their mineralized landmarks fade from repeated use, and crack and crumble with weathering, has there been discussion of their use and disposition. For example, some scholars have framed the replacement of historical teaching collections as a “remedy” to offer “improved” and “representative” collections, and to replace them with “authentic skeletons”^23^. While the dedication to replace teaching skeletal collections with remains from willed donors is admirable, attention is also required for the ethical and equitable disposition of Indian skeletal remains, which currently do not always match protocols of commemoration and cremation for contemporary (primarily White) donor bodies. Other scholars have suggested that the continued use of historical skeletons, particularly those obtained before the 1985 ban, is ethical in professional settings^36^, although this ignores and upholds unethical and colonial practices of collection and use. Other institutions have extended the ethical mandates of NAGPRA law intended for the repatriation of Native American ancestors to non-Native American human remains that have limited provenance information, including recommendations such as communal reburial for teaching collection remains with varied acquisition histories^40^. However, remains from South Asia likely include individuals of both Hindu and Muslim faith, and the former faith quite strongly favors the cremation of the dead. Similarly, it should not be considered ethical to repatriate South Asian remains to other descendant groups, for example, under other Indigenous protection laws.

Clearly, my positionality as both a professional bioarchaeologist and a person of South Asian descent, informs my judgment. I recall the moment when I first became cognizant of the exclusive use of Indian skeletons for teaching. Just over three decades ago, I enrolled in my first undergraduate human osteology class, finding my passion as a biological anthropologist. While I recognized I was the only person of color in the undergraduate major, I still went in with naive excitement of making it in. As we began to lay out our assigned teaching skeletons for the year, the instructor explained to us why the sexual dimorphism of the skull was limited in the collection, because the skeletons were from India. While everyone nodded at the information given and went back to continue to busily work away learning the name of bones, time stopped for me as I looked around the room and realized that the 30 or so bodies strewn across the tables were ***all*** Indian. I realized I had more in common with the skeletons on the table than my fellow students. I did not yet have the language of decolonization to question the power dynamics of scientific authority^41^ or the voice to recognize my subjectivity as a researcher and subject of structural inequality^42^. What I did have was a feeling of shame. Shame that I was born in Canada as the result of the sheer will and educational opportunities of my immigrant parents, that were themselves raised by my grandparents that came from the same abject poverty and grade school-level education as my teaching skeleton. Shame that I would go on to use my study skeleton, and similar ones, that year and in the many years that followed in my training without acknowledgement of their origins, although I made a point to not teach my own students with remains from India or without contemporary standards of informed consent.

I do not think there is one best way to stop using or dispose of anatomical skeletons from India, or even if we should stop using them. Further, it is not feasible or appropriate to ask to repatriate these ancestors to a country that, as of 2021, has a population of 1.4 billion and faced the disposal of 10.23 million dead in 2021^43^, but that also does not implement a uniform Anatomy Act across its states and continues to allow the acquisition of anatomical bodies primarily from unclaimed bodies^44^. What is key, is that *who* should decide what happens to these skeletal remains needs to be South Asian descendants both abroad and in India, but with the expectation that we cannot simply expect compliance with the newly found ethics of the Western world. In the case of the unknown dead, where there can be no individual informed consent, the obligation of researchers are to the descendants, whether they be lineal descendants with historical ties or representative of the local social community^45,46^. The African Burial Ground Project in New York City established a model of ethical engagement with descendant groups, the clientage model, that served the interests of the descendant community for dignified treatment, study, and disposition^45,47^ Similar ethical conversation and collaboration with South Asian descendant representatives and communities will take time to develop. While there is a rush to put a moratorium on the use of historical anatomical teaching collections, we also need to listen to the voices of South Asian decedents in local communities that may want continued use, partial moratoriums, or the informed use of collections whereby students who study the skeletons are educated on the full history and collection of bodies from India. Contemporary medical schools that use willed bodies have started to inform students of the personal history, demographics, cause of death, the first names of donors, and in some schools, even meet families of the donors^48,49^. Similar tools that utilize pedagogical empathy, even learning and sharing limited known demographic information, should be put in place for the use of historic South Asian anatomical skeletons to aid in reclaiming their personhood. The Western world created and stoked an industry of bodily commodification for over a century, we should now create space for the development of a uniquely South Asian bioethical response that is not simply rooted in colonial guilt. We are obligated to historicize the people that were systematically made into anatomical objects, to reflect on our role in upholding the necropolitical aims that created and continue to create these skeletal collections, and to search for appropriate ways to return their dignity.
